# Molecular epidemiological surveillance for non-tuberculous mycobacterial pulmonary disease: a single-center prospective cohort study

**DOI:** 10.1128/spectrum.00436-25

**Published:** 2025-08-21

**Authors:** Kazuki Hashimoto, Kiyoharu Fukushima, Yuki Matsumoto, Haruko Saito, Atsushi Funauchi, Nanako Hamada, Takayuki Niitsu, Yuko Abe, June Yamauchi, Tadayoshi Nitta, Daisuke Motooka, Takuro Nii, Takanori Matsuki, Kazuyuki Tsujino, Keisuke Miki, Atsushi Kumanogoh, Shota Nakamura, Hiroshi Kida

**Affiliations:** 1 Department of Respiratory Medicine and Clinical Immunology, Osaka University Graduate School of Medicine 198269 , Suita, Osaka, Japan; 2 Department of Infection Metagenomics, Genome Information Research Center, Research Institute for Microbial Diseases, Osaka University 13013, Suita, Osaka, Japan; 3 Laboratory of Host Defense, World Premier Institute Immunology Frontier Research Center (WPI-IFReC), Osaka University 13013, Suita, Osaka, Japan; 4 Department of Clinical laboratory, National Hospital Organization, Osaka Toneyama Medical Centre, Toyonaka, Osaka, Japan; 5 Department of Respiratory Medicine, National Hospital Organization, Osaka Toneyama Medical Centre 13013, Toyonaka, Osaka, Japan; National Institute of Immunolog, New Delhi, India

**Keywords:** core genome multi-locus sequence typing, digital variable number tandem repeat, genotyping, next-generation sequencing, non-tuberculous mycobacteria

## Abstract

Bacterial species cultured from sputum change during treatment or observation for non-tuberculous mycobacterial pulmonary disease; however, strain-level changes remain unrecognized. Variable number tandem repeat typing is a standard technique for strain identification; nonetheless, its labor-intensive and time-consuming nature limits routine clinical use. Therefore, we aimed to elucidate species-subspecies and strain dynamics in non-tuberculous mycobacteria and develop a simple sequence-based strain-level determination method. We performed a single-center prospective cohort study of 112 patients with non-tuberculous mycobacterial pulmonary disease. Whole-genome sequencing was performed on two sputum samples collected at enrollment and at the end of follow-up, followed by variable number tandem repeat (VNTR) typing. We also developed a simple long-read sequencing-based digital VNTR (dVNTR) typing method and evaluated its efficacy. Our results demonstrate that core genome multi-locus sequencing typing revealed species/subspecies changes in 13 patients (11.6%); VNTR typing detected strain changes in 16 patients (14.3%) without species/subspecies changes. Overall, pathogen shifts occurred in 29 patients (shift [+] group, 25.9%), whereas 83 had no detectable pathogen shift (shift [−] group, 74.1%). Interestingly, macrolide and amikacin susceptibility changed in both groups, but resistance remained higher in shift (−) patients. dVNTR results aligned with those of conventional VNTR typing. In conclusion, since susceptibility factors remain unclear, routine species/subspecies identification and molecular typing, such as VNTR, are optimal for patient care. Core genome multi-locus sequencing typing with a dVNTR identified pathogen shifts, innovating non-tuberculous mycobacterial pulmonary disease management.

**Clinical Trials:**

This study is registered with UMIN as UMIN 000056067.

**IMPORTANCE:**

Pulmonary non-tuberculous mycobacterial disease is a chronic infection in which the causative pathogens may change at the species, subspecies, or strain level over time. Accurate tracking of these changes is essential for optimizing treatment; however, conventional clinical practice lacks efficient methods for monitoring such dynamics. Our study revealed pathogen changes in approximately one-quarter of patients over 1.5 years, prompting the development of a novel surveillance system that integrates next-generation sequencing for both species-subspecies identification and strain-level molecular epidemiology. This innovation enables real-time monitoring of pathogen dynamics, allowing clinicians to promptly adjust treatment strategies and improve patient care through more informed decision-making.

## INTRODUCTION

Non-tuberculosis mycobacteria (NTM) comprise approximately 200 species (1), and their associated infectious diseases have significantly increased globally, becoming a major public health concern (2). While the precise identification of NTM species is essential for effective treatment, it often necessitates a combination of multiple tests, which can be time-consuming, labor-intensive, and expensive. Consequently, the number of species identification tests conducted for a single patient with NTM pulmonary disease (NTM-PD) is generally limited in daily clinical practice despite recognition of their critical importance (3). In our previous studies, we developed a novel method, called MGIT-Seq, for the comprehensive identification of NTM at the subspecies level through core genome multi-locus sequence typing (cgMLST) analyzed using *mlstverse* software and for the prediction of macrolide and amikacin (AMK) resistance based on previously reported mutations in *rrl*, *rrs*, and *erm(41*) using DNA extracted from culture-positive broths and sequenced with a MinION portable sequencer. Our prospective cohort study demonstrated that MGIT-Seq achieved 99.1% accuracy in species-level identification and successfully determined 84.5% of isolates at the subspecies level, with predicted macrolide and AMK resistance showing high consistency with conventional drug susceptibility tests (specificities of 97.6 and 100.0%, respectively). Moreover, MGIT-Seq has been shown to save time and labor, making it suitable for implementation in busy clinical settings (3, 4).

The question is whether MGIT-Seq is implemented in routine clinical practice and if it has the potential to revolutionize the diagnosis and treatment of NTM-PD. There are several unresolved issues in treating NTM-PD. A pivotal concern is that although prior studies have shown that recurrence may result from either relapse or reinfection, necessitating distinct treatment approaches (5), these two causes are seldom differentiated in everyday clinical practice due to technical and resource constraints. Despite these acknowledged difficulties, there is a dearth of comprehensive longitudinal studies that investigate the frequency of strain changes and the effects of NTM infections over time. Thus, our goal was to develop a feasible method to overcome these diagnostic obstacles specifically to monitor the dynamics of strain changes in clinical environments.

In this study, we established a prospective cohort of patients with NTM-PD to examine transitions in mycobacterial strains using cgMLST and variable number tandem repeat (VNTR) typing. Recognizing that more patients experience a pathogen shift than expected, we developed a long-read sequencing-based digital VNTR (dVNTR) typing method and integrated it into our *mlstverse* analysis platform. We aimed to establish a comprehensive analytical method using the MinION portable sequencer that combines two functions: (i) species-subspecies identification through cgMLST and (ii) strain identification by dVNTR. The streamlined workflow of this all-in-one approach is intended to facilitate implementation in routine clinical settings.

## MATERIALS AND METHODS

### Study design and population

**Fig 1:**
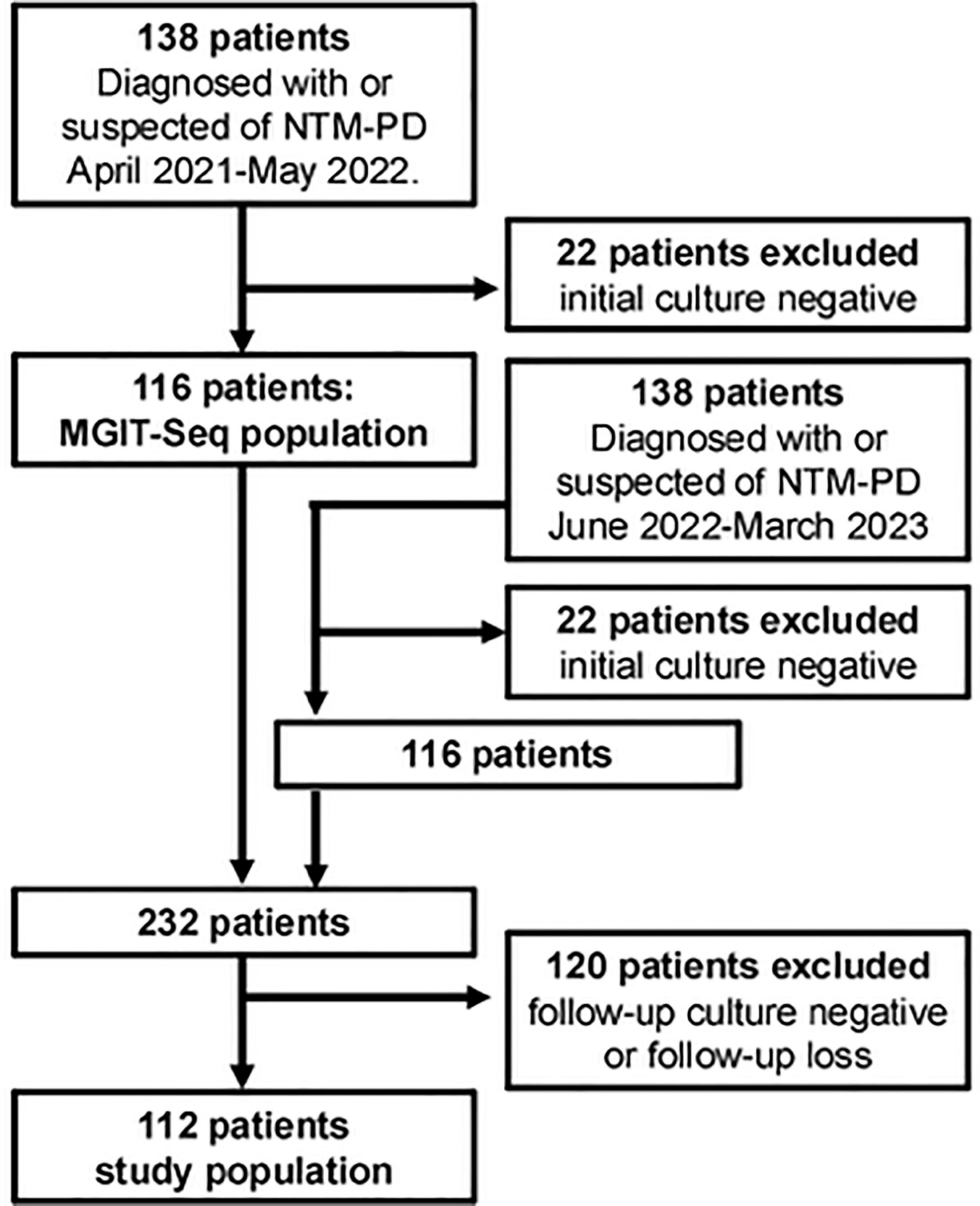
Flow diagram of patient enrollment and analysis in this study.

In this single-center prospective cohort study, between April 2021 and May 2022, we recruited 138 patients diagnosed with or suspected of having NTM-PD, in accordance with international guidelines (6), who provided written consent to participate in the Toneyama NTM cohort. After excluding 22 patients with initial culture-negative results, 116 patients (MGIT-Seq population) were included in our previous study (Fig. 1) (3). Between June 2022 and March 2023, we recruited another 138 patients, of whom 22 were excluded because their initial cultures were negative. The remaining 116 patients were integrated into the MGIT-Seq population to form a cohort of 232 patients. Follow-up cultures were obtained at least 6 months after the initial culture, and the latest available isolates near the end of the observation period were selected. We excluded 120 patients who had negative follow-up cultures (Fig. 1). The final cohort consisted of 112 patients, and all patients met the diagnostic criteria for NTM-PD.

### Baseline characteristics

Baseline clinical information collected at initial culture included age, sex, body mass index, acid-fast bacillus smear status (positive or negative), anti-glycopeptidolipid-core IgA antibody levels, and the presence of cavities on CT. Anti-glycopeptidolipid-core IgA antibody levels were defined as positive when they exceeded 0.7 U/mL (7). During the observation period, the use of multidrug combination therapy for 6 months or longer was recorded. At both the initial and follow-up sampling, species-subspecies identification using cgMLST, drug susceptibility testing, and VNTR typing were performed and compared. Additionally, dVNTR typing was performed only on polymerase chain reaction (PCR)-free genomes.

### cgMLST analysis

For genomic analysis of mycobacterial isolates, DNA was extracted from MGIT or Ogawa egg cultures using the NucleoSpin Microbial DNA Kit (Takara Bio, Kusatsu, Japan) or the DNeasy PowerSoil Pro Kit (QIAGEN, Baarn, Netherlands). The sequencing library was prepared using the Ligation Sequencing Kit V14 (SQK-LSK114, Oxford Nanopore Technologies, Oxford, UK) and the PCR-free Native Barcoding Kit 96 V14 (SQK-NBD114.96, Oxford Nanopore Technologies, Oxford, UK). It was then sequenced using the P2 solo instrument (PRO-SEQ002, Oxford Nanopore Technologies, Oxford, UK) with flow cells R10.4.1 (FLO-PRO114M, Oxford Nanopore Technologies, Oxford, UK) for long-read sequencing.

As previously described and reported (3), samples with sequence codes beginning with CW (Table S1) were sequenced directly from MGIT-positive broths. For these samples, the library was prepared using the Rapid PCR Barcoding Kit (SQK-RPB004, ONT). Sequencing was then performed on the MinION sequencer (MIN-101B, ONT) with flowcells R9.4.1 (FLO-MIN106D, ONT).

Sequence data were analyzed using cgMLST with the mlstverse software (Department of Infectious Disease Metagenomics, Osaka University, Suita, Japan) (4).

### Drug susceptibility test

To evaluate antimicrobial susceptibility, the minimum inhibitory concentrations (MICs) for *Mycobacterium avium* complex (MAC) were determined with the broth dilution method using BrothMIC SGM (Kokuto Pharmaceutical Industrial Co., Ltd., Tokyo, Japan), which complies with Clinical and Laboratory Standards Institute (CLSI) M24S 2nd edition. The breakpoints for clarithromycin (CLR) were susceptible at 8 µg/mL, intermediate at 16 µg/mL, and resistant at ≥32 µg/mL. The breakpoints for amikacin were susceptible at 16 µg/mL, intermediate at 32 µg/mL, and resistant at ≥64 µg/mL. The MICs for *M. abscessus* (*MAB*) were determined using BrothMIC RGM (Kokuto Pharmaceutical Industrial Co., Ltd.) following the CLSI M24 guidelines (8). The breakpoints for CLR were susceptible at 2 µg/mL, intermediate at 4 µg/mL, and resistant at 8 µg/mL. The breakpoints for AMK were susceptible at 16 µg/mL, intermediate at 32 µg/mL, and resistant at ≥64 µg/mL.

### VNTR protocol

For VNTR typing, we employed distinct primer sets for each species: 15 VNTR loci for *M. avium* (9), 16 for *M. intracellulare* (10), and nine for *MAB* (11). The PCR amplification conditions included an initial denaturation at 94°C for 2 min, followed by 35 cycles of denaturation at 98°C for 10 s, annealing at 68°C for 30 s, and extension at 72°C for 1 min. Electrophoresis was performed on 2% agarose gel at 40 V for 50 min. A 100 bp DNA size marker (Takara Bio, Kusatsu, Japan) was simultaneously electrophoresed on the samples for size comparison. For each VNTR locus, the final PCR product sizes were estimated based on electrophoresis results, and the number of tandem repeats was determined by rounding to the nearest integer value. These were manually checked to ensure accuracy. A difference of one or more repeats at any locus was considered a different VNTR type (12, 13).

### dVNTR protocol

PCR-based library preparation in next-generation sequencing may introduce biases in VNTR region amplification (14); therefore, only PCR-free genomes were used. Genome sequences were initially assembled using the *Flye* software (15). The VNTR regions were then identified by aligning the primer sets for *M. avium*, *M. intracellulare*, and *MAB* used in conventional VNTR typing (9–11) using the EMBOSS *primersearch* software, which identifies primer binding sites in the assembled genome sequences (16). For each detected VNTR locus, the product sizes were calculated. The number of tandem repeats was determined by rounding to the nearest integer. As with the conventional VNTR, any variation of one or more repeats at any locus was considered a different VNTR type (Fig. S1).

### Statistical analysis

For statistical analyses, continuous data are reported as medians with interquartile ranges and categorical data as frequencies and percentages. Comparisons between two groups for continuous variables were conducted using the Wilcoxon rank-sum test, whereas group differences for categorical variables were evaluated using Fisher’s exact test.

## RESULTS

**Fig 2:**
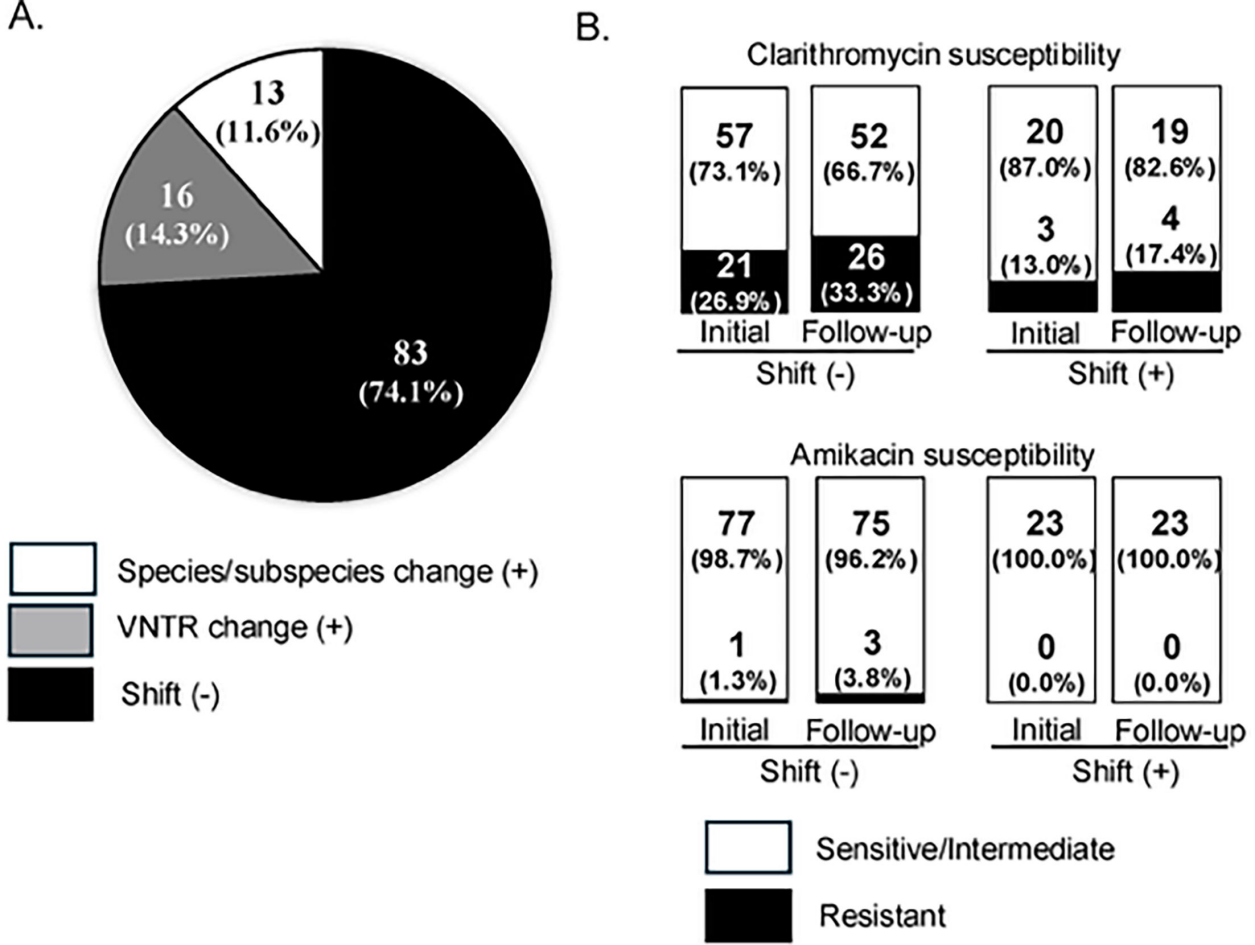
Comparison of NTM between initial and follow-up culture. (**A**) Number (proportion) of patients showing a change in bacterial species/subspecies, patients showing a change in VNTR, and patients showing neither of these changes. (**B**) Number (proportion) of patients with clarithromycin and amikacin sensitive/intermediate or resistant among patients who showed pathogen shift (Shift [+]) or did not show pathogen shift (Shift [−]). NTM, non-tuberculosis mycobacteria; VNTR, variable number tandem repeat.

Among the 112 patients with culture-positive specimens, the median time from the enrollment period to the follow-up period conducted at least 6 months later was 1.5 years (interquartile range, 1.1–1.9). cgMLST identified 65 isolates of *M. avium* subsp. *hominissuis* (58.0%), 25 of *M. intracellulare* subsp. *intracellulare* (21.9%), five of *M. intracellulare* subsp. *chimera* (4.4%), six of *M. abscessus* subsp. *abscessus* (5.3%), and four of *M. abscessus* subsp. *massiliense* (3.5%) at enrollment (Table 1). During the follow-up period, a change in the species/subspecies of NTM occurred in 13 patients (11.6%) (Fig. 2A). Some patients exhibited a shift from *M. avium* subsp. *hominissuis* to *M. intracellulare* subsp. *intracellulare*, whereas others shifted from *M. intracellulare* subsp. *intracellulare* to *M. avium* subsp. *hominissuis*. We could not find any specific trend in the tendency of certain species/subspecies of bacteria to shift (Table 1). Of these patients, three had cavitary lesions in their lungs, and seven were undergoing treatment for NTM-PD with multidrug chemotherapy (Table 1).

**TABLE 1:** Clinical characteristics^*c*^

	Total *n* = 112	Pathogen shift		*P*-value^*a*^
		(+) *n* = 29	(−) *n* = 83	
Sex, female	81 (72.3)^*b*^	20 (69.0)	61 (73.5)	0.637
Age (years)	73 (67–80)	77 (69–81)	72 (66–79)	0.102
Body mass index (kg/m^2^)	18.9 (16.9–20.8)	18.5 (16.3–21.3)	19.0 (16.9–20.7)	0.948
Cavity	53 (47.3)	9 (31.0)	44 (53.0)	0.052
AFB stain-positive	46 (41.1)	35 (42.2)	11 (37.9)	0.827
Anti-GPL-core IgA antibody-positive (*n* = 111)	93/111 (83.8)	22/28 (78.6)	71/83 (85.5)	0.387
Species-subspecies by cgMLST				
*MA* subsp. *hominissuis*	65 (58.0)	17 (58.6)	48 (57.8)	1.000
*MI* subsp. *intracellulare*	25 (21.9)	8 (27.6)	17 (20.5)	0.445
*MI* subsp. *chimera*	5 (4.4)	0 (0.0)	5 (6.0)	0.325
*MAB* subsp. *abscessus*	6 (5.3)	1 (3.4)	5 (6.0)	1.000
*MAB* subsp. *massiliense*	4 (3.5)	1 (3.4)	3 (3.6)	1.000
*M. peregrinum*	1 (0.9)	1 (3.4)	0 (0.0)	0.259
*M. paragordonae*	1 (0.9)	1 (3.4)	0 (0.0)	0.259
*M. lentiflavum*	1 (0.9)	0 (0.0)	1 (1.2)	1.000
*M. szulgai*	1 (0.9)	0 (0.0)	1 (1.2)	1.000
*M. mugogenicum*	1 (0.9)	0 (0.0)	1 (1.2)	1.000
*M. kansasii*	1 (0.9)	0 (0.0)	1 (1.2)	1.000
*M. simiae*	1 (0.9)	0 (0.0)	1 (1.2)	1.000
Macrolide resistance in MAC and *MAB*				
Initial (*n* = 101)	24/101 (23.8)	3/23 (13.0)	21/78 (26.9)	0.265
Follow-up (*n* = 100)	30/101 (29.7)	4/23 (17.4)	26/78 (33.3)	0.196
Amikacin resistance				
Initial (*n* = 101)	1/101 (1.0)	0/23 (0.0)	1/78 (1.3)	1.000
Follow-up (*n* = 101)	3/101 (3.0)	0/23 (0.0)	3/78 (3.8)	1.000
Multidrug therapy (>6 months)	51 (45.5)	13 (44.8)	38 (45.8)	1.000
Observation period (years)	1.5 (1.1–1.9)	1.6 (1.0–2.0)	1.5 (1.2–1.7)	0.623

^*a*^ Differences among continuous variables were assessed using the Wilcoxon rank-sum test; differences among categorical variables were assessed using Fisher’s exact test, as appropriate.

^*b*^ Data are presented as *n* (%) or median (interquartile range).

^*c*^ AFB, acid-fast bacillus; GPL, glycopeptidolipid; cgMLST, core genome multi-locus sequence typing;* MA*, *Mycobacterium avium*; *MI*, *Mycobacterium intracellulare*; *MAB*, *Mycobacterium abscessus*; *M*., *Mycobacterium*.

**Fig 3:**
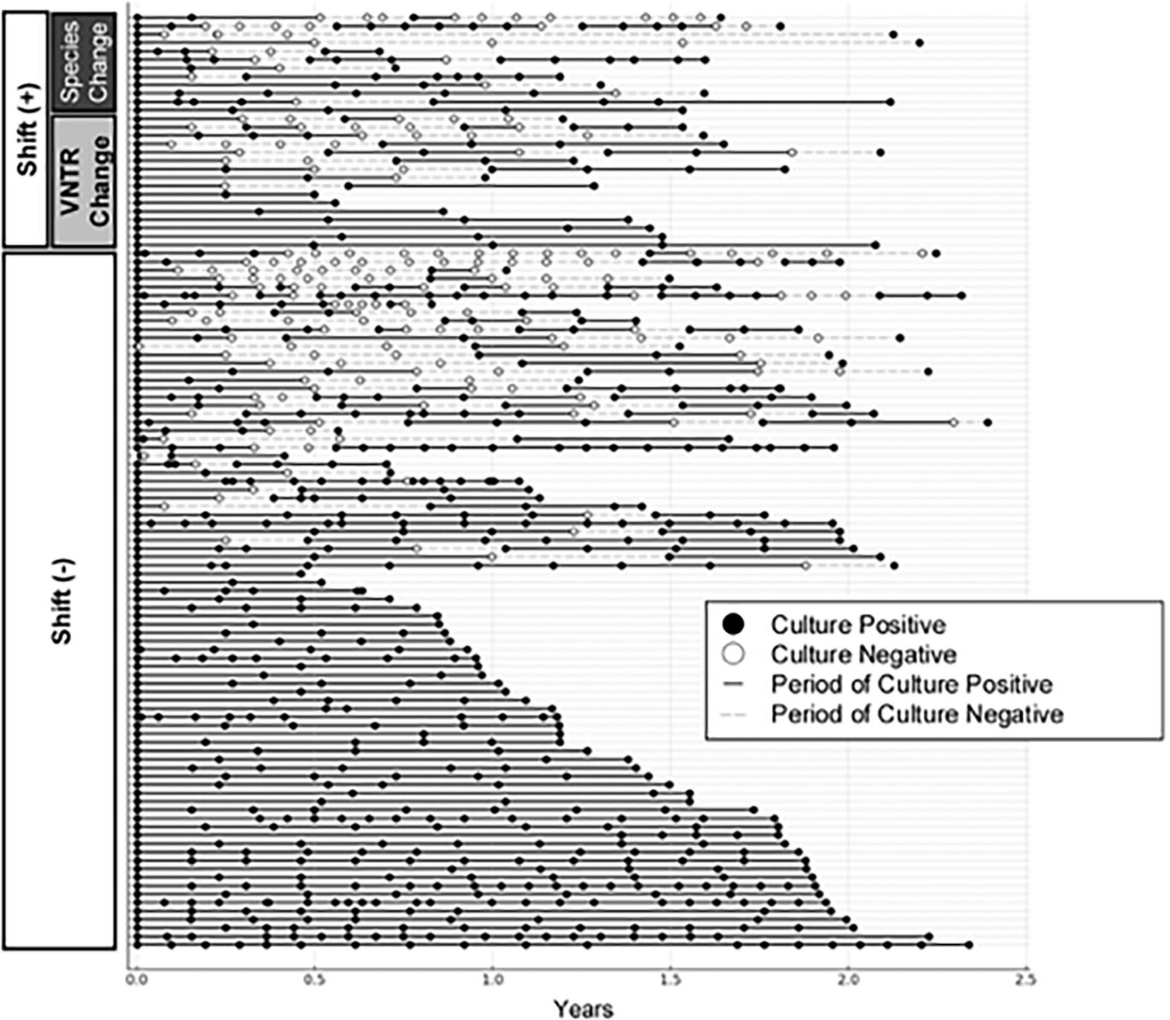
Timeline of culture results and species or VNTR changes during the study period. VNTR, variable number tandem repeat.

Among the remaining 99 patients in whom the species/subspecies did not change, VNTR typing was performed on 94 patients in whom MAC and *MAB* were detected in their sputum. Among these, 16 showed VNTR-type changes (Fig. 2A). Similar to patients who showed a change in bacterial species/subspecies, it is thought that these patients experienced a shift in the infecting NTM bacterial strains (shift [+] group, *n* = 29) (Table 2). The infection persisted with the same strain in the remaining 83 patients (shift [−] group, *n* = 83). Subsequently, we conducted a comparative analysis of the clinical characteristics between the shift (+) and shift (−) groups (Table 1). Although the difference was not statistically significant, non-cavitary nodular bronchiectatic (NB) disease was more common in the shift (+) group (20/29, 69.0%) than in the shift (−) group (39/83, 47.0%) (Table 1). Temporal changes in sputum culture status and shifts in NTM infection are depicted in Fig. 3. The shift in NTM infection occurred irrespective of whether culture positivity continued or was interrupted during follow-up.

**TABLE 2:** Patients with pathogen shift (+)^*a*^

#	Initial	Follow-up	MDT	Cavity
Species/subspecies change (+)				
1	*MA* subsp. *hominissuis*	*MAB* subsp. *abscessus*	+	−
2	*MA* subsp. *hominissuis*	*MAB* subsp. *massiliense*	+	+
3	*MA* subsp. *hominissuis*	*MI* subsp. *chimaera*	+	−
4	*MA* subsp. *hominissuis*	*MI* subsp. *intracellulare*	−	−
5	*MA* subsp. *hominissuis*	*MI* subsp. *intracellulare*	−	−
6	*MA* subsp. *hominissuis*	*M. lentiflavum*	+	−
7	*MA* subsp. *hominissuis*	*M. mageritense*	−	−
8	*MA* subsp. *hominissuis*	*M. paragordonae*	+	+
9	*MI* subsp. *intracellulare*	*MA* subsp. *hominissuis*	−	−
10	*MI* subsp. *intracellulare*	*MA* subsp. *hominissuis*	+	−
11	*MI* subsp. *intracellulare*	*M. mageritense*	+	+
12	*M. paragordonae*	*MI* subsp. *chimaera*	−	−
13	*M. peregrinum*	*M. chelonae*	−	−
Species/subspecies change (−), VNTR change (+)				
14	*MA* subsp. *hominissuis*	*MA* subsp. *hominissuis*	−	−
15	*MA* subsp. *hominissuis*	*MA* subsp. *hominissuis*	+	+
16	*MA* subsp. *hominissuis*	*MA* subsp. *hominissuis*	+	+
17	*MA* subsp. *hominissuis*	*MA* subsp. *hominissuis*	−	+
18	*MA* subsp. *hominissuis*	*MA* subsp. *hominissuis*	+	−
33	*MA* subsp. *hominissuis*	*MA* subsp. *hominissuis*	−	−
36	*MA* subsp. *hominissuis*	*MA* subsp. *hominissuis*	−	+
38	*MA* subsp. *hominissuis*	*MA* subsp. *hominissuis*	−	−
37	*MA* subsp. *hominissuis*	*MA* subsp. *hominissuis*	+	+
92	*MI* subsp. *intracellulare*	*MI* subsp. *intracellulare*	−	−
83	*MI* subsp. *intracellulare*	*MI* subsp. *intracellulare*	+	−
81	*MI* subsp. *intracellulare*	*MI* subsp. *intracellulare*	−	−
84	*MI* subsp. *intracellulare*	*MI* subsp. *intracellulare*	+	−
82	*MI* subsp. *intracellulare*	*MI* subsp. *intracellulare*	−	−
108	*MAB* subsp. *massiliense*	*MAB* subsp. *massiliense*	−	+
106	*MAB* subsp. *massiliense*	*MAB* subsp. *massiliense*	−	−

^*a*^ MDT, multidrug therapy; MA, *Mycobacterium avium*; MI, *Mycobacterium intracellulare*; MAB, *Mycobacterium abscessus*; VNTR, variable number of tandem repeats; +, present; −, absent.

In the cohort of 112 patients, drug susceptibility tests (DSTs) for CLR and AMK both at enrollment and at least 6 months later were performed in 101 patients (23 in the shift [+] group, 78 in the shift [−] group) with MAC and *MAB* detected in their sputum. The percentage of patients with CLR resistance was higher in the shift (−) group (21/78, 26.9%) than in the shift (+) group (3/23, 13.0%) (*P* = 0.265) (Table 1; Fig. 2B). Additionally, the percentage of CLR resistance in the shift (−) and shift (+) groups increased during follow-up (26/78 [33.3%] and 4/23 [17.4%], respectively) (Table 1; Fig. 2B). AMK resistance was less common, with 1.3% (1/78) of the initial samples and 3.8% (3/78) of the follow-up samples in the shift (−) group. Notably, AMK resistance was not observed in the shift (+) group (Table 1; Fig. 2B). The change in DST was observed at a similar frequency in the shift (−) group (11/78, 14.1%) and the shift (+) group (3/23, 13.0%) (*P* = 1.000) (Table 3).

**TABLE 3:** Patients who showed changes in drug susceptibility^*a*^

No.	Initial	Follow-up	VNTR change	Drug	Change
Shift (+) group					
1	*MA* subsp. *hominissuis*	*MAB* subsp. *abscessus*	N.A.	CLR	S→IR
18	*MA* subsp. *hominissuis*	*MA* subsp. *hominissuis*	+	CLR	S→R
37	*MA* subsp. *hominissuis*	*MA* subsp. *hominissuis*	+	CLR	R→S
Shift (−) group					
88	*MI* subsp. *intracellulare*	*MI* subsp. *intracellulare*	−	CLR AMK	S→R S→R
49	*MA* subsp. *hominissuis*	*MA* subsp. *hominissuis*	−	CLR AMK	S→R R→I
89	*MI* subsp. *intracellulare*	*MI* subsp. *intracellulare*	−	CLR	S→R
21	*MA* subsp. *hominissuis*	*MA* subsp. *hominissuis*	−	CLR	S→R
47	*MA* subsp. *hominissuis*	*MA* subsp. *hominissuis*	−	CLR	S→R
55	*MA* subsp. *hominissuis*	*MA* subsp. *hominissuis*	−	CLR	S→R
61	*MA* subsp. *hominissuis*	*MA* subsp. *hominissuis*	−	CLR	S→R
71	*MA* subsp. *hominissuis*	*MA* subsp. *hominissuis*	−	CLR	R→S
104	*MI* subsp. *intracellulare*	*MI* subsp. *intracellulare*	−	CLR	R→S
54	*MA* subsp. *hominissuis*	*MA* subsp. *hominissuis*	−	AMK	S→R
58	*MA* subsp. *hominissuis*	*MA* subsp. *hominissuis*	−	AMK	S→R

^*a*^ MA, *Mycobacterium avium*; MI, *Mycobacterium intracellulare*; MAB, *Mycobacterium abscessus*; N.A., not available; +, changed; −, unchanged; CLR, clarithromycin; AMK, amikacin; VNTR, variable number of tandem repeats; S, sensitive; I, intermediate; R, resistant.

**Fig 4:**
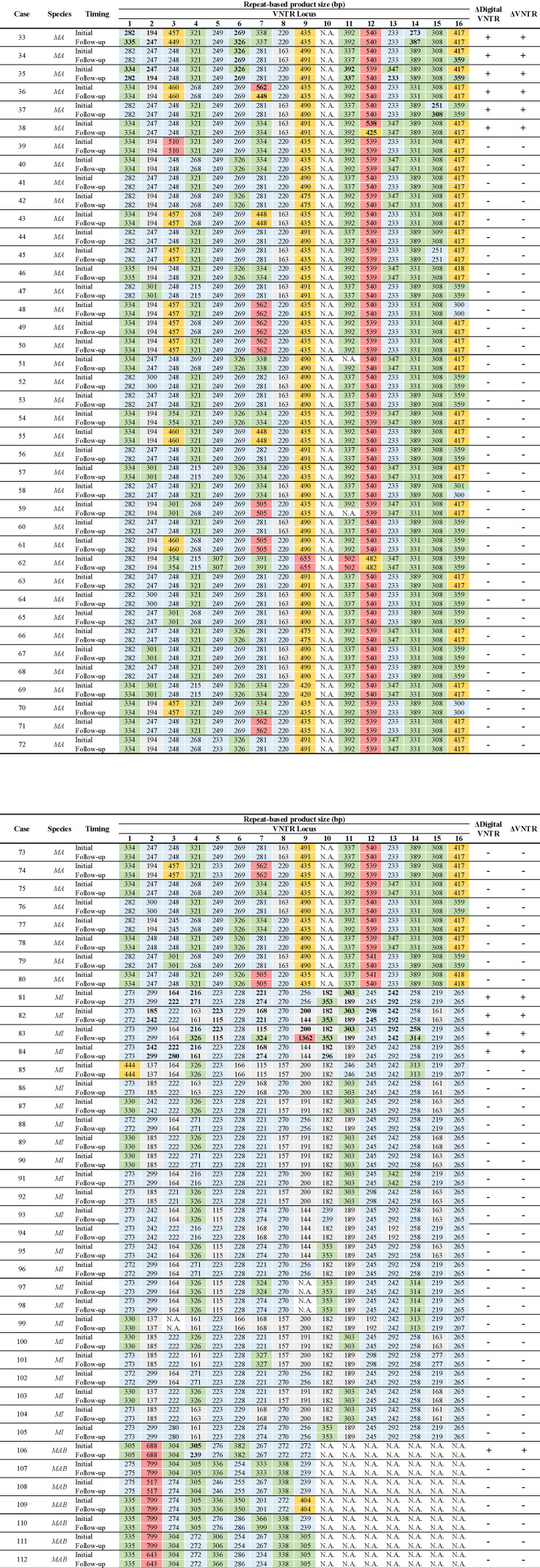
The results of the digital VNTR (dVNTR) analysis for 80 pairs of non-tuberculous mycobacteria from initial and follow-up cultures are shown. For *Mycobacterium avium* (*MA*), *Mycobacterium intracellulare* (*MI*), and *Mycobacterium abscessus* (*MAB*), the number of nucleotides at 15, 16, and 9 VNTR loci, respectively, were analyzed. The presence (+) or absence (−) of differences in dVNTR score was perfectly consistent with those identified by conventional VNTR (ΔVNTR). VNTR, variable number tandem repeat.

In this study, we recognized that distinguishing between shift (+) and shift (−) groups was clinically significant. Therefore, we developed a simple long-read sequencing-based digital VNTR (dVNTR) typing method that enabled VNTR analysis in routine clinical practice. Briefly, DNA sequences read by a long-read sequencer were assembled, and the numbers of tandem repeats between primer pairs used in conventional VNTR were quantified as numerical values (Fig. S1). To demonstrate the accuracy of dVNTR in diagnosing VNTR variations, we compared the results of dVNTR with those of conventional VNTR. Among the 80 patients, conventional VNTR identified 79 cases with the same VNTR types and four with different types. dVNTR produced identical results, with sensitivity, specificity, positive predictive value, and negative predictive value all calculated to be 100.0% (Fig. 4).

## DISCUSSION

To the best of our knowledge, this is the first prospective cohort study that followed NTM isolates during treatment or observation and used the high-resolution methods of VNTR and cgMLST, demonstrating a high frequency of pathogen shifts at the strain level among patients with NTM-PD. Over a 1.5-year period, 25.9% of patients experienced a pathogen shift, highlighting the dynamic nature of NTM-PD. Lim et al. retrospectively identified 133 patients with NTM-PD who underwent two or more species identification tests for NTM isolates during treatment or observation and found that 37 patients (27.8%) had multiple species (17). Lee et al. also retrospectively reviewed data from 164 patients with NTM-PD who started treatment and found that 54 patients (32.9%) had shifts in NTM species (18). However, these retrospective studies have focused only on changes at the species level (17, 18). Furthermore, there is a bias in that species identification tests are typically performed only in cases of poor treatment response, recurrence, or changes in the characteristics of the cultured strains. In this study, we enrolled consecutive patients and regularly performed comprehensive species/subspecies identification tests using cgMLST, followed by strain identification tests using VNTR. Our patients were typical individuals with NTM-PD, whom we routinely manage in our clinical practice. In this study, the shift (−) group had more patients with cavitary lesions and drug-resistant conditions than the shift (+) group. This finding is significant because cavitation and drug resistance are key prognostic factors associated with poor outcomes in NTM-PD patients (19, 20). The higher prevalence of drug resistance in the shift (−) group suggests that persistent infection with a single strain under antibiotic pressure promotes resistance development. This interpretation is consistent with previous reports where reinfection cases exhibited lower macrolide resistance (21, 22). Wallace et al. demonstrated that polyclonal NTM infections primarily occur in the NB type, while monoclonal infections are prevalent in the fibrocavitary type, which is linked to adverse outcomes (23). Similarly, in bronchiectasis, akin to NB-type NTM-PD, a reduction in microbiome diversity has been correlated with increased disease severity, more frequent and severe exacerbations, and a higher mortality risk (24). Consequently, a dominant infection by a single strain may contribute to disease progression in NTM-PD patients. More intensive strategies, such as long-duration treatment, adding aminoglycosides, or surgical resection, may be considered for patients with single-strain infections. In contrast, the reassessment of environmental exposures or host-related risk factors may be warranted for cases of reinfection with a different strain. Regular sputum culture tests, along with bacterial strain identification, can help clarify this. Recently, Lee et al. analyzed pretreatment and on-treatment clinical isolates of MAC-PD by PCR-based MLST analysis of five housekeeping genes, reporting genetically distinct strains within the same species at least once in approximately 25% of patients’ on-treatment isolates (18). Since the clinical characteristics of patients susceptible to pathogen shifts have not been established, periodic species/subspecies identification testing and molecular typing, such as VNTR, would be optimal for infection control of patients. Our newly developed long-read sequencing-based dVNTR demonstrated equivalent or superior typing resolution compared to conventional VNTR analysis while dramatically simplifying the workflow (Fig. S2). Although conventional VNTR typing offers clearer results and remains as the simplest method relative to restriction fragment length polymorphism (9), rep-PCR (25), and MLST (26), it still necessitates approximately 1 day and significant effort due to the single-plex analysis of 9–16 loci, which complicates its routine use in clinical practice (10). In recent years, sequence-based technologies have become increasingly accessible and are now broadly applied to mycobacterial identification (3, 27). Early next-generation sequencing technologies were limited to short reads (50–300 bp), which could not span entire VNTR regions up to 1,000 bp, preventing accurate assembly. The breakthrough came with the emergence of long-read sequencing (over thousands bp) (28), which now enables dVNTR with high accuracy (Fig. S3). Our novel long read-based dVNTR method overcomes these challenges through three key innovations: (i) multiplexed VNTR loci analysis with single-base resolution, exceeding the capabilities of conventional gel electrophoresis-based estimates; (ii) a rapid turnaround time of approximately 3 h from sequencing initiation to results; and (iii) an automated workflow requiring minimal bioinformatics expertise (Fig. S2). Therefore, high-throughput strain-level typing has become a clinical reality because our dVNTR method analyzes multiple samples in approximately 3 h, replacing the conventional process, which required one day per patient. It allows for the distinction between relapse and reinfection in recurrent cases and may also support routine surveillance by identifying persistent strains associated with drug resistance. This approach can help clinicians make more informed and personalized treatment decisions for patients with NTM-PD.

This study has some limitations. First, our cohort primarily consisted of patients with NB-type NTM-PD, with fewer patients having FC type, reflecting the distribution observed in routine clinical practice in Japan. Additional studies are needed to clarify the epidemiology and strain dynamics in other disease types, especially in the FC type, which exhibits lower clonal diversity compared to the NB type (23). Second, the median interval between sample collections was 1.5 years, and the sample size was limited to a single-center cohort. Longer observation periods and validation in a larger cohort are needed to fully understand the strain dynamics in chronic NTM-PD. Third, we did not evaluate the relationship between environmental factors and strain variations. Environmental exposure may affect strain changes; further detailed investigations on this aspect are warranted.

In conclusion, this prospective cohort study clarified the dynamic transition of NTM strains in patients with NTM-PD. Our sequence-based all-in-one analytical method using cgMLST and digital VNTR enabled the comprehensive identification of NTM at the strain level, the significance of which was demonstrated in our cohort study.  

## Data Availability

The data sets supporting the conclusions of this study are included in this article. The data sets generated and analyzed here are available from the corresponding author upon reasonable request. The raw sequencing data supporting the findings of this study have been deposited in NCBI and DDBJ under BioProject accession numbers PRJDB12894, PRJDB19189, and PRJNA1274183.

## SUPPLEMENTAL MATERIAL

Supplemental figures spectrum.00436-25-s0001.pdf Fig. S1 to S3.

Table S1 spectrum.00436-25-s0002.docx Data mapping table.

ASM does not own the copyrights to Supplemental Material that may be linked to, or accessed through, an article. The authors have granted ASM a non-exclusive, world-wide license to publish the Supplemental Material files. Please contact the corresponding author directly for reuse.
