# Supplementary material for: Molecular epidemiological surveillance for non-tuberculous mycobacterial pulmonary disease: a single-center prospective cohort study
Source: Microbiol Spectr. 2025 Aug 21;13(10):e00436-25. doi: 10.1128/spectrum.00436-25 (PMC12502804; doi:10.1128/spectrum.00436-25)

Fig. S1 Illustration of the genome of non-tuberculous mycobacteria (NTM) and the concept of VNTR typing. NTM genomes are circular and contain 9 to 16 VNTR loci, depending on the species. These VNTR loci are composed of repetitive units, and the number of units in each locus determines the genotype of the strain. This figure uses *Mycobacterium intracellulare* as an example, which contains 16 VNTR loci. For instance, in "Locus 1," the base pair length of the VNTR locus differs between the initial and follow-up samples due to differences in the number of repetitive units, indicating that they represent distinct strains. The total length of each VNTR locus is calculated as the sum of the flanking region (used for PCR amplification) and the length contributed by the repetitive units, which is obtained by multiplying the number of units by the length of a single repetitive unit.

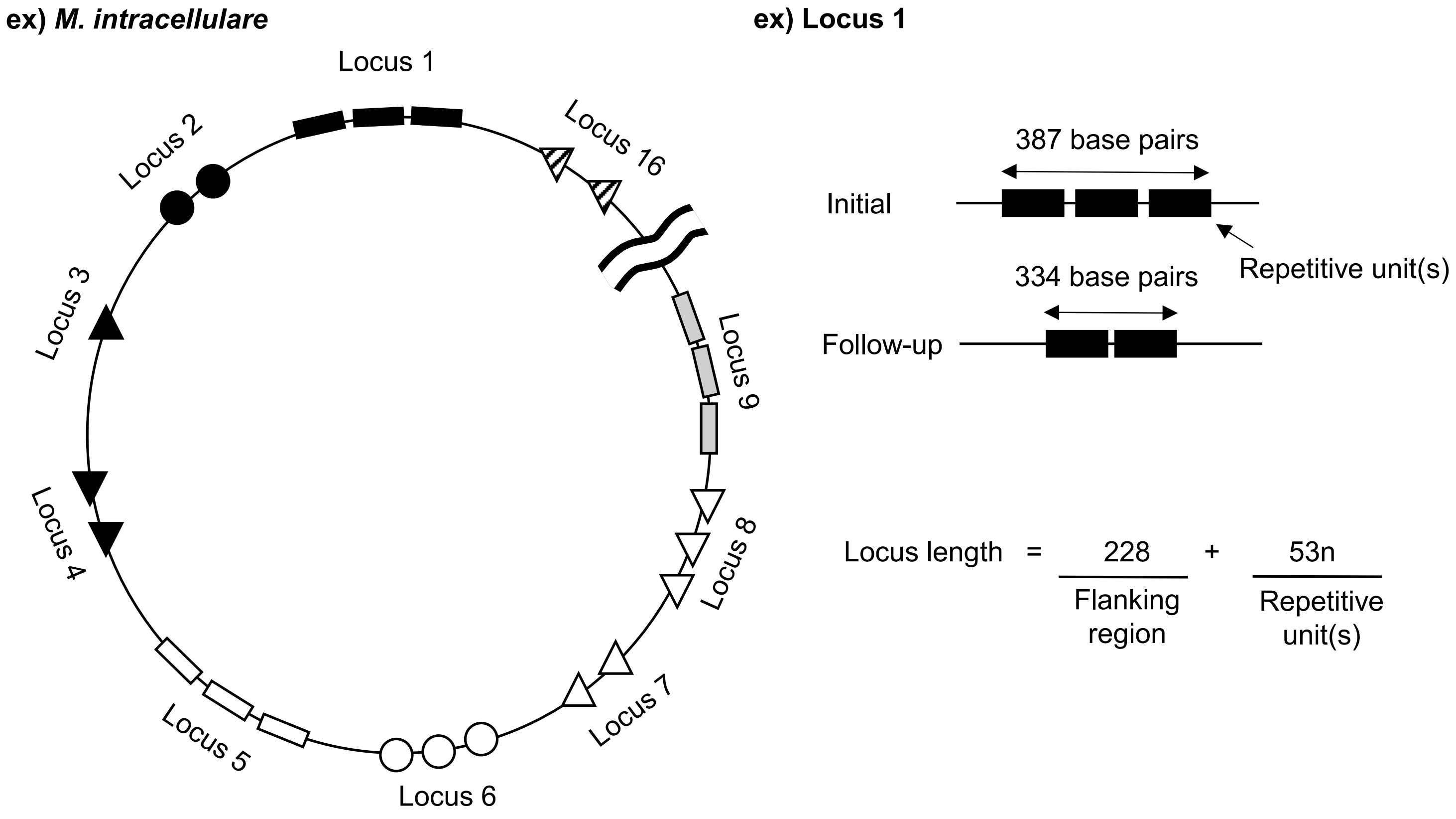

Fig. S2 The protocol differences between VNTR and dVNTR. Conventional VNTR requires PCR for each of the 9–16 VNTR loci, using specific primer pairs designed for each locus, applied to both initial and follow-up samples. The resulting PCR products are analyzed individually through gel electrophoresis to calculate base pair lengths, making the process labor-intensive and time-consuming. In contrast, dVNTR enables whole genome sequencing of multiple samples with one click, automatically calculating VNTR region lengths.

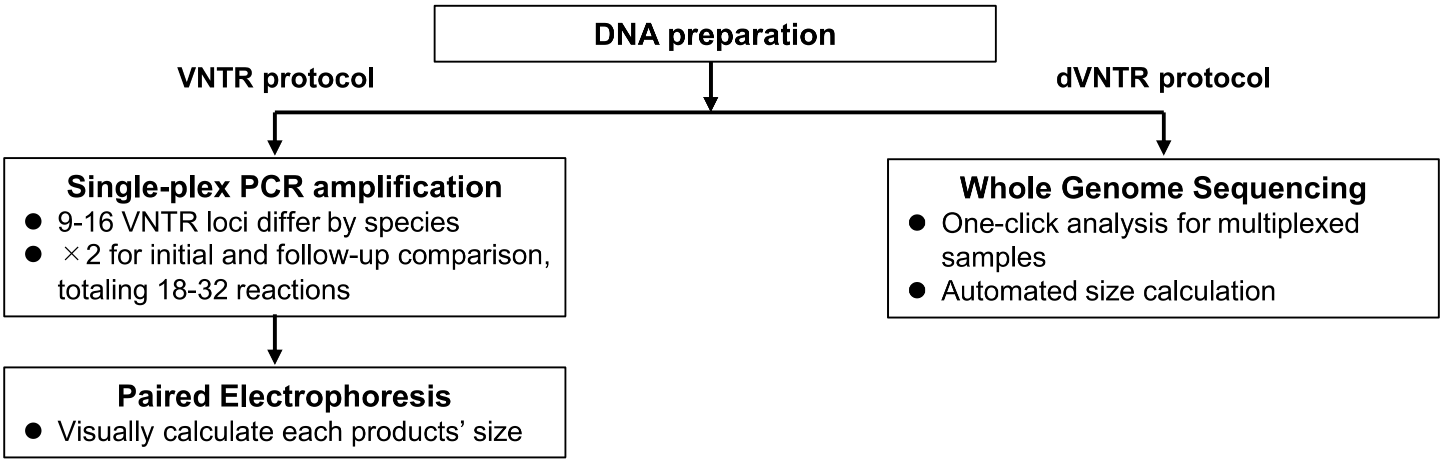

Fig. S3 Illustration of the differences in VNTR region coverage between short-read and long-read sequencing. Short-read sequencing often fails to fully cover the entire VNTR region, making it difficult to accurately determine the total length of the repetitive sequences. In contrast, long-read sequencing can cover the entire VNTR region, enabling precise measurement of the total length of the repetitive sequences.

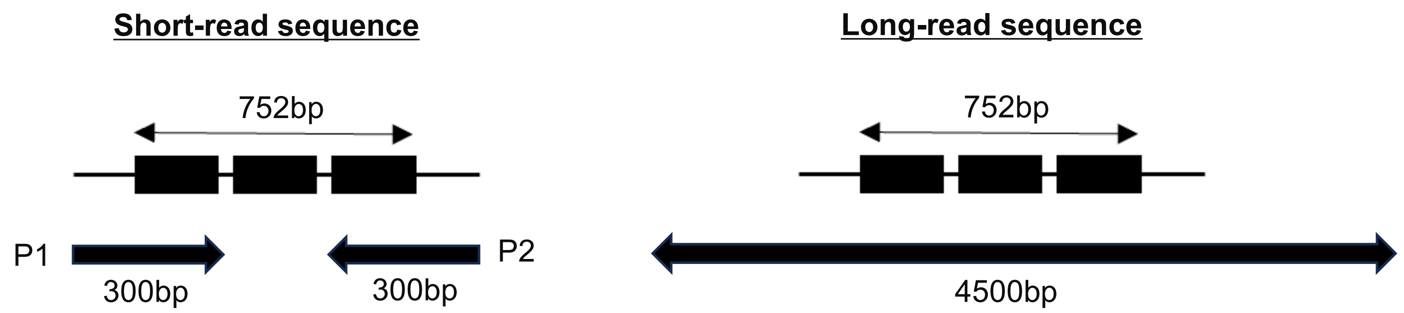

Supplement: Supplemental figures — Fig. S1 to S3. [file spectrum.00436-25-s0001.pdf]
