# Supplementary material for: Molecular epidemiological surveillance for non-tuberculous mycobacterial pulmonary disease: a single-center prospective cohort study
Source: Microbiol Spectr. 2025 Aug 21;13(10):e00436-25. doi: 10.1128/spectrum.00436-25 (PMC12502804; doi:10.1128/spectrum.00436-25)
Supplement: Table S1 — Data mapping table. [file spectrum.00436-25-s0002.docx]

**Table S1. Data mapping table**

| No. | Timing | Code | BioProject Accession | Run Accession |
| --- | --- | --- | --- | --- |
| 1 | initial | CW25785 | PRJDB12894 | DRR337673 |
|  | follow-up | GA68622 | PRJDB19189 | DRR629194 |
| 2 | initial | CW23763 | PRJDB12894 | DRR337637 |
|  | follow-up | GA68603 | PRJDB19189 | DRR629196 |
| 3 | initial | GA61912 | PRJDB19189 | DRR629277 |
|  | follow-up | GA61911 | PRJDB19189 | DRR629313 |
| 4 | initial | CW24349 | PRJDB12894 | DRR337649 |
|  | follow-up | GA61958 | PRJDB19189 | DRR629201 |
| 5 | initial | CW25816 | PRJDB12894 | DRR395242 |
|  | follow-up | GA61963 | PRJDB19189 | DRR629235 |
| 6 | initial | CW24405 | PRJDB12894 | DRR337662 |
|  | follow-up | GA61960 | PRJDB19189 | DRR629260 |
| 7 | initial | CW28309 | PRJDB12894 | DRR395285 |
|  | follow-up | GA68648 | PRJDB19189 | DRR629258 |
| 8 | initial | CW28335 | PRJDB12894 | DRR395286 |
|  | follow-up | GA68639 | PRJDB19189 | DRR629259 |
| 9 | initial | CW28330 | PRJDB12894 | DRR395272 |
|  | follow-up | GA68633 | PRJDB19189 | DRR629250 |
| 10 | initial | GA70180 | PRJDB19189 | DRR629303 |
|  | follow-up | GA70197 | PRJDB19189 | DRR629339 |
| 11 | initial | GA70166 | PRJDB19189 | DRR629290 |
|  | follow-up | GA70183 | PRJDB19189 | DRR629326 |
| 12 | initial | CW25814 | PRJDB12894 | DRR337669 |
|  | follow-up | GA61936 | PRJDB19189 | DRR629215 |
| 13 | initial | CW28224 | PRJDB12894 | DRR395262 |
|  | follow-up | GA61959 | PRJDB19189 | DRR629246 |
| 14 | initial | CW24160 | PRJDB12894 | DRR337650 |
|  | follow-up | GA68607 | PRJDB19189 | DRR629202 |
| 15 | initial | CW28302 | PRJDB12894 | DRR395269 |
|  | follow-up | GA61949 | PRJDB19189 | DRR629249 |
| 16 | initial | CW28307 | PRJDB12894 | DRR395276 |
|  | follow-up | GA68635 | PRJDB19189 | DRR629252 |
| 17 | initial | CW28451 | PRJDB12894 | DRR395304 |
|  | follow-up | GA68649 | PRJDB19189 | DRR629269 |
| 18 | initial | CW28338 | PRJDB12894 | DRR395292 |
|  | follow-up | GA68642 | PRJDB19189 | DRR629263 |
| 19 | initial | CW23775 | PRJDB12894 | DRR337635 |
|  | follow-up | GA68602 | PRJDB19189 | DRR629194 |
| 20 | initial | CW24348 | PRJDB12894 | DRR337647 |
|  | follow-up | GA68606 | PRJDB19189 | DRR629200 |
| 21 | initial | CW24345 | PRJDB12894 | DRR337653 |
|  | follow-up | GA61941 | PRJDB19189 | DRR629205 |
| 22 | initial | CW24158 | PRJDB12894 | DRR337642 |
|  | follow-up | GA61956 | PRJDB19189 | DRR629225 |
| 23 | initial | CW28217 | PRJDB12894 | DRR395252 |
|  | follow-up | GA61962 | PRJDB19189 | DRR629242 |
| 24 | initial | CW28333 | PRJDB12894 | DRR395283 |
|  | follow-up | GA68638 | PRJDB19189 | DRR629256 |
| 25 | initial | CW23780 | PRJDB12894 | DRR337634 |
|  | follow-up | GA61946 | PRJDB19189 | DRR629228 |
| 26 | initial | CW24360 | PRJDB12894 | DRR337659 |
|  | follow-up | GA61965 | PRJDB19189 | DRR629211 |
| 27 | initial | CW28278 | PRJDB12894 | DRR395278 |
|  | follow-up | GA61964 | PRJDB19189 | DRR629268 |
| 28 | initial | CW28235 | PRJDB12894 | DRR395250 |
|  | follow-up | GA68646 | PRJDB19189 | DRR629240 |
| 29 | initial | CW28221 | PRJDB12894 | DRR395259 |
|  | follow-up | GA68629 | PRJDB19189 | DRR629244 |
| 30 | initial | GA61906 | PRJDB19189 | DRR629281 |
|  | follow-up | GA61905 | PRJDB19189 | DRR629317 |
| 31 | initial | GA70167 | PRJDB19189 | DRR629291 |
|  | follow-up | GA70184 | PRJDB19189 | DRR629327 |
| 32 | initial | GA70175 | PRJDB19189 | DRR629299 |
|  | follow-up | GA70192 | PRJDB19189 | DRR629335 |
| 33 | initial | GA91924 | PRJNA1274183 | SRR33905611 |
|  | follow-up | GA61957 | PRJDB19189 | DRR629206 |
| 34 | initial | GA91931 | PRJNA1274183 | SRR33905608 |
|  | follow-up | GA61938 | PRJDB19189 | DRR629214 |
| 35 | initial | GA91955 | PRJNA1274183 | SRR33905592 |
|  | follow-up | GA68628 | PRJDB19189 | DRR629241 |
| 36 | initial | GA61896 | PRJDB19189 | DRR629274 |
|  | follow-up | GA61895 | PRJDB19189 | DRR629310 |
| 37 | initial | GA61898 | PRJDB19189 | DRR629278 |
|  | follow-up | GA61897 | PRJDB19189 | DRR629314 |
| 38 | initial | GA70170 | PRJDB19189 | DRR629294 |
|  | follow-up | GA70187 | PRJDB19189 | DRR629330 |
| 39 | initial | GA91922 | PRJNA1274183 | SRR33905564 |
|  | follow-up | GA68608 | PRJDB19189 | DRR629203 |
| 40 | initial | GA91923 | PRJNA1274183 | SRR33905563 |
|  | follow-up | GA68609 | PRJDB19189 | DRR629204 |
| 41 | initial | GA91929 | PRJNA1274183 | SRR33905562 |
|  | follow-up | GA61945 | PRJDB19189 | DRR629212 |
| 42 | initial | GA91930 | PRJNA1274183 | SRR33905609 |
|  | follow-up | GA68612 | PRJDB19189 | DRR629213 |
| 43 | initial | GA91933 | PRJNA1274183 | SRR33905607 |
|  | follow-up | GA68615 | PRJDB19189 | DRR629217 |
| 44 | initial | GA91934 | PRJNA1274183 | SRR33905606 |
|  | follow-up | GA61942 | PRJDB19189 | DRR629218 |
| 45 | initial | GA91935 | PRJNA1274183 | SRR33905605 |
|  | follow-up | GA68616 | PRJDB19189 | DRR629219 |
| 46 | initial | GA91936 | PRJNA1274183 | SRR33905604 |
|  | follow-up | GA61935 | PRJDB19189 | DRR629220 |
| 47 | initial | GA91937 | PRJNA1274183 | SRR33905603 |
|  | follow-up | GA68617 | PRJDB19189 | DRR629221 |
| 48 | initial | GA91938 | PRJNA1274183 | SRR33905602 |
|  | follow-up | GA68618 | PRJDB19189 | DRR629222 |
| 49 | initial | GA91940 | PRJNA1274183 | SRR33905601 |
|  | follow-up | GA68620 | PRJDB19189 | DRR629224 |
| 50 | initial | GA91946 | PRJNA1274183 | SRR33905600 |
|  | follow-up | GA68644 | PRJDB19189 | DRR629230 |
| 51 | initial | GA91947 | PRJNA1274183 | SRR33905598 |
|  | follow-up | GA68624 | PRJDB19189 | DRR629231 |
| 52 | initial | GA91948 | PRJNA1274183 | SRR33905597 |
|  | follow-up | GA68645 | PRJDB19189 | DRR629232 |
| 53 | initial | GA91950 | PRJNA1274183 | SRR33905596 |
|  | follow-up | GA61932 | PRJDB19189 | DRR629234 |
| 54 | initial | GA91951 | PRJNA1274183 | SRR33905595 |
|  | follow-up | GA61948 | PRJDB19189 | DRR629236 |
| 55 | initial | GA91952 | PRJNA1274183 | SRR33905594 |
|  | follow-up | GA68626 | PRJDB19189 | DRR629237 |
| 56 | initial | GA91953 | PRJNA1274183 | SRR33905593 |
|  | follow-up | GA61952 | PRJDB19189 | DRR629238 |
| 57 | initial | GA91958 | PRJNA1274183 | SRR33905591 |
|  | follow-up | GA61933 | PRJDB19189 | DRR629243 |
| 58 | initial | GA91960 | PRJNA1274183 | SRR33905590 |
|  | follow-up | GA68631 | PRJDB19189 | DRR629247 |
| 59 | initial | GA91968 | PRJNA1274183 | SRR33905589 |
|  | follow-up | GA68636 | PRJDB19189 | DRR629254 |
| 60 | initial | GA91969 | PRJNA1274183 | SRR33905587 |
|  | follow-up | GA68637 | PRJDB19189 | DRR629255 |
| 61 | initial | GA91975 | PRJNA1274183 | SRR33905586 |
|  | follow-up | GA68641 | PRJDB19189 | DRR629262 |
| 62 | initial | GA91977 | PRJNA1274183 | SRR33905585 |
|  | follow-up | GA68643 | PRJDB19189 | DRR629264 |
| 63 | initial | GA91979 | PRJNA1274183 | SRR33905584 |
|  | follow-up | GA61944 | PRJDB19189 | DRR629266 |
| 64 | initial | GA91980 | PRJNA1274183 | SRR33905583 |
|  | follow-up | GA61953 | PRJDB19189 | DRR629267 |
| 65 | initial | GA61904 | PRJDB19189 | DRR629273 |
|  | follow-up | GA61903 | PRJDB19189 | DRR629309 |
| 66 | initial | GA61918 | PRJDB19189 | DRR629276 |
|  | follow-up | GA61917 | PRJDB19189 | DRR629312 |
| 67 | initial | GA61930 | PRJDB19189 | DRR629283 |
|  | follow-up | GA61929 | PRJDB19189 | DRR629319 |
| 68 | initial | GA61922 | PRJDB19189 | DRR629284 |
|  | follow-up | GA61921 | PRJDB19189 | DRR629320 |
| 69 | initial | GA61914 | PRJDB19189 | DRR629285 |
|  | follow-up | GA61913 | PRJDB19189 | DRR629321 |
| 70 | initial | GA61908 | PRJDB19189 | DRR629286 |
|  | follow-up | GA61907 | PRJDB19189 | DRR629322 |
| 71 | initial | GA61902 | PRJDB19189 | DRR629287 |
|  | follow-up | GA61901 | PRJDB19189 | DRR629323 |
| 72 | initial | GA70168 | PRJDB19189 | DRR629292 |
|  | follow-up | GA70185 | PRJDB19189 | DRR629328 |
| 73 | initial | GA70169 | PRJDB19189 | DRR629293 |
|  | follow-up | GA70186 | PRJDB19189 | DRR629329 |
| 74 | initial | GA70171 | PRJDB19189 | DRR629295 |
|  | follow-up | GA70188 | PRJDB19189 | DRR629331 |
| 75 | initial | GA70172 | PRJDB19189 | DRR629296 |
|  | follow-up | GA70189 | PRJDB19189 | DRR629332 |
| 76 | initial | GA70174 | PRJDB19189 | DRR629298 |
|  | follow-up | GA70191 | PRJDB19189 | DRR629334 |
| 77 | initial | GA70176 | PRJDB19189 | DRR629300 |
|  | follow-up | GA70193 | PRJDB19189 | DRR629336 |
| 78 | initial | GA70179 | PRJDB19189 | DRR629302 |
|  | follow-up | GA70196 | PRJDB19189 | DRR629338 |
| 79 | initial | GA70181 | PRJDB19189 | DRR629304 |
|  | follow-up | GA70198 | PRJDB19189 | DRR629340 |
| 80 | initial | GA70182 | PRJDB19189 | DRR629305 |
|  | follow-up | GA70199 | PRJDB19189 | DRR629341 |
| 81 | initial | GA91943 | PRJNA1274183 | SRR33905588 |
|  | follow-up | GA68621 | PRJDB19189 | DRR629227 |
| 82 | initial | GA91954 | PRJNA1274183 | SRR33905566 |
|  | follow-up | GA68627 | PRJDB19189 | DRR629239 |
| 83 | initial | GA91961 | PRJNA1274183 | SRR33905599 |
|  | follow-up | GA61951 | PRJDB19189 | DRR629248 |
| 84 | initial | GA91967 | PRJNA1274183 | SRR33905577 |
|  | follow-up | GA61947 | PRJDB19189 | DRR629253 |
| 85 | initial | GA91918 | PRJNA1274183 | SRR33905570 |
|  | follow-up | GA61937 | PRJDB19189 | DRR629195 |
| 86 | initial | GA91919 | PRJNA1274183 | SRR33905582 |
|  | follow-up | GA61931 | PRJDB19189 | DRR629197 |
| 87 | initial | GA91920 | PRJNA1274183 | SRR33905581 |
|  | follow-up | GA68605 | PRJDB19189 | DRR629198 |
| 88 | initial | GA91921 | PRJNA1274183 | SRR33905580 |
|  | follow-up | GA61961 | PRJDB19189 | DRR629199 |
| 89 | initial | GA91925 | PRJNA1274183 | SRR33905579 |
|  | follow-up | GA68610 | PRJDB19189 | DRR629207 |
| 90 | initial | GA91926 | PRJNA1274183 | SRR33905578 |
|  | follow-up | GA68611 | PRJDB19189 | DRR629208 |
| 91 | initial | GA91927 | PRJNA1274183 | SRR33905576 |
|  | follow-up | GA61955 | PRJDB19189 | DRR629209 |
| 92 | initial | GA91939 | PRJNA1274183 | SRR33905610 |
|  | follow-up | GA68619 | PRJDB19189 | DRR629223 |
| 93 | initial | GA91942 | PRJNA1274183 | SRR33905575 |
|  | follow-up | GA61939 | PRJDB19189 | DRR629226 |
| 94 | initial | GA91949 | PRJNA1274183 | SRR33905574 |
|  | follow-up | GA68625 | PRJDB19189 | DRR629233 |
| 95 | initial | GA91965 | PRJNA1274183 | SRR33905573 |
|  | follow-up | GA68634 | PRJDB19189 | DRR629251 |
| 96 | initial | GA91970 | PRJNA1274183 | SRR33905572 |
|  | follow-up | GA68647 | PRJDB19189 | DRR629257 |
| 97 | initial | GA91978 | PRJNA1274183 | SRR33905571 |
|  | follow-up | GA61934 | PRJDB19189 | DRR629265 |
| 98 | initial | GA61910 | PRJDB19189 | DRR629272 |
|  | follow-up | GA61909 | PRJDB19189 | DRR629308 |
| 99 | initial | GA61926 | PRJDB19189 | DRR629275 |
|  | follow-up | GA61925 | PRJDB19189 | DRR629311 |
| 100 | initial | GA61928 | PRJDB19189 | DRR629279 |
|  | follow-up | GA61927 | PRJDB19189 | DRR629315 |
| 101 | initial | GA61900 | PRJDB19189 | DRR629282 |
|  | follow-up | GA61899 | PRJDB19189 | DRR629318 |
| 102 | initial | GA70164 | PRJDB19189 | DRR629288 |
|  | follow-up | GA70200 | PRJDB19189 | DRR629324 |
| 103 | initial | GA70165 | PRJDB19189 | DRR629289 |
|  | follow-up | GA70201 | PRJDB19189 | DRR629325 |
| 104 | initial | GA70173 | PRJDB19189 | DRR629297 |
|  | follow-up | GA70190 | PRJDB19189 | DRR629333 |
| 105 | initial | GA70178 | PRJDB19189 | DRR629301 |
|  | follow-up | GA70195 | PRJDB19189 | DRR629337 |
| 106 | initial | GA61924 | PRJDB19189 | DRR629270 |
|  | follow-up | GA61923 | PRJDB19189 | DRR629306 |
| 107 | initial | GA91928 | PRJNA1274183 | SRR33905569 |
|  | follow-up | GA61954 | PRJDB19189 | DRR629210 |
| 108 | initial | GA91932 | PRJNA1274183 | SRR33905565 |
|  | follow-up | GA68614 | PRJDB19189 | DRR629216 |
| 109 | initial | GA91959 | PRJNA1274183 | SRR33905568 |
|  | follow-up | GA68630 | PRJDB19189 | DRR629245 |
| 110 | initial | GA91974 | PRJNA1274183 | SRR33905567 |
|  | follow-up | GA68640 | PRJDB19189 | DRR629261 |
| 111 | initial | GA61916 | PRJDB19189 | DRR629271 |
|  | follow-up | GA61915 | PRJDB19189 | DRR629307 |
| 112 | initial | GA61920 | PRJDB19189 | DRR629280 |
|  | follow-up | GA61919 | PRJDB19189 | DRR629316 |
